# Micronization using combined alkaline protease hydrolysis and high-speed shearing homogenization for improving the functional properties of soy protein isolates

**DOI:** 10.1186/s40643-022-00565-9

**Published:** 2022-07-25

**Authors:** Junyu Hao, Zhuchi Zhang, Ming Yang, Yongli Zhang, Tao Wu, Rui Liu, Wenjie Sui, Min Zhang

**Affiliations:** 1 Sate Key Laboratory of Food Nutrition and Safety, Food Biotechnology Engineering Research Center of Ministry of Education, Tianjin University of Science & Technology, Tianjin, 300457 China; 2 Tianjin Agricultural University, Tianjin, 300384 China; 3 China-Russia Agricultural Processing Joint Laboratory, Tianjin Agricultural University, Tianjin, 300392 People’s Republic of China

**Keywords:** Alkaline protease, Hydrolysis degree, Soy protein isolate, High-speed shearing homogenization, Functional properties

## Abstract

The present study aimed to investigate the functional properties of soybean protein isolate (SPI) treated with alkaline protease and high-speed shearing homogenization. Alkaline protease-hydrolyzed SPIs that were characterized by varying degrees of hydrolysis between 0 and 6% were treated with high-speed shearing homogenization to obtain different micro-particulate proteins. The results showed that this combined treatment could significantly reduce the particle size of SPI by markedly degrading the structure of both the 7S and 11S subunits, thereby resulting in a significantly reduced content of β-sheet and β-turn structures. The surface hydrophobicity increased considerably for samples with hydrolysis below the threshold of 2% and then declined gradually above this threshold. Furthermore, the combination of hydrolysis and homogenization significantly improved the emulsion stability of SPI hydrolysates. It also significantly improved the foaming properties of SPI. These results demonstrated that alkaline protease hydrolysis combined with high-speed shearing homogenization represents a promising approach for improving the functional and structural properties of SPI.

**Graphical Abstract:**

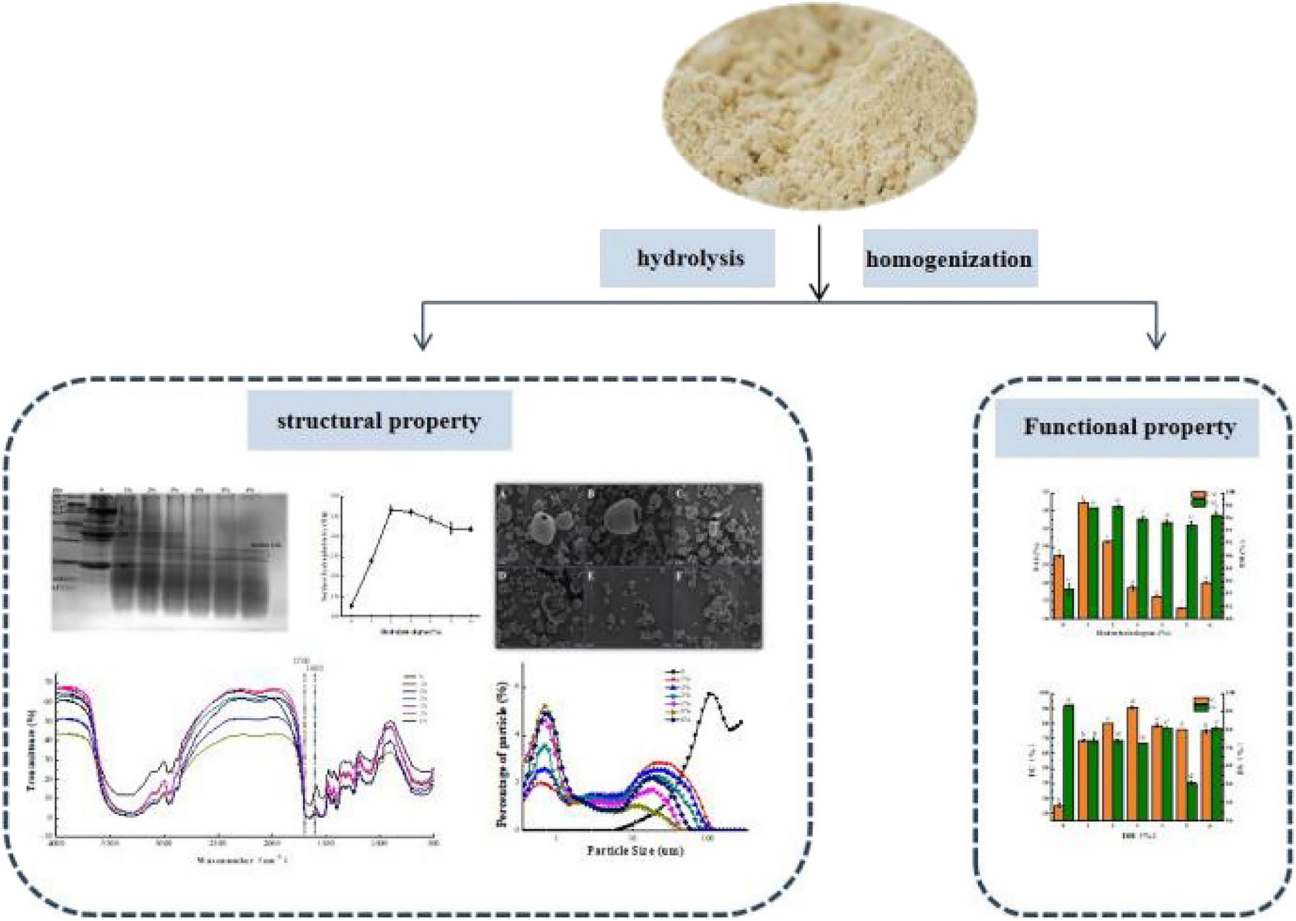

## Introduction

Soybean protein isolate (SPI) is a whole-protein food additive produced from low-temperature solvent-extracted soybean meal. It has long been regarded as an ideal alternative to animal protein due to its desirable amino acid profile, lack of cholesterol (Ma et al. 2020a), and more cost-effective production method (Pozdnyakov et al. 2022). Being an amphiphilic protein, soybean protein isolate is used as an emulsifier to generate oil-in-water emulsions with strong diffusion and adsorption capabilities to stabilize the surface of oil droplets (Yan et al. 2021). However, some severe processing conditions, such as acid precipitation and high temperatures; can cause the denaturation of SPI, thereby resulting in its poor solubility, poor structural properties, and other undesirable functional properties (Yang et al. 2018).

To improve upon the quality and application value of SPI, micronization technology has become widely used to enhance the structural and functional characteristics of SPI. This process can also lead to the production of short peptide sequences with various bioactivities (Ashaolu 2020; Hsieh et al. 2022). Micro-particulate proteins are formed by altering the way proteins aggregate, and because of the involution of hydrophobic groups, there is a low frequency with which other groups interact with them. Therefore, adding high-density SPI protein particles to the food system not only improves the texture and taste of the system but also increases both the overall protein and bioactive polypeptide contents of foods (Li et al. 2021a; Sha et al. 2020).

At present, there are several micro-particulate protein production techniques available, including double emulsification, mechanical processing, phase separation, and the combining with other macromolecules (Silva and Chandrapala 2021; Zhang et al. 2022). Previous studies have found that, compared with other techniques, the process of modifying proteins by hydrolysis is relatively easy to control, producing few by-products with mild hydrolysis reaction conditions, and hence considered to be relatively resource-efficient (Li et al. 2021b; Song et al. 2018). This process of hydrolysis is an effective approach for producing hypoallergenic soy hydrolysates that combine several desirable functional properties (Meinlschmidt et al. 2016). Hydrolysis can also create a variety of bioactive peptides which play a positive role in the prevention and treatment of diseases such as hypertension and cancer, as well as the physiological burden of oxidative damage (Álvarez-Viñas et al. 2021; Guan et al. 2018; Wang et al. 2017; Wu et al. 2021). High-speed shear homogenization is now extensively used in food engineering. Its treatment used alone has relatively low efficiency for improving the qualities of SPI. Many studies have found that combined treatments can improve functional properties of consumer protein. For instance, high-pressure homogenization treatment in conjunction with enzymatic hydrolysis could modify the properties of peanut protein isolate and increase the antioxidant activities of its hydrolysates (Dong et al. 2011). The treatment has also been used to improve the functional properties of oyster protein isolates (Yu et al. 2018). The combined treatment of extrusion pretreatment with controlled enzymatic hydrolysis using pancreatin was shown to improve the emulsifying capability of SPIs (Chen et al. 2011). To the best of our knowledge, no studies have yet assessed the utility of this combined method of hydrolysis with high-speed shearing homogenization. Thus, research on improving the structural and functional properties of SPIs by this treatment method might be of great utility in improving the manufacturing of foods containing them.

The objective of this study was to use hydrolysis and high-speed shear homogenization to reduce the particle size of micro-particulate SPI and to explore the resulting changes in their processing performance and microstructures. The results can then be used to provide a better understanding of the usage of the combined treatment in food processing. The findings would also be significant for guiding the development of functional SPI products in a theoretical sense. Approximately 90% of SPIs are storage proteins with globular structures comprising mainly 7S (β-conglycinin) and 11S (glycinin) globulins, and this study intends to develop a highly effective method for the functional modification of such globular proteins.

## Materials and methods

### Materials

SPI was purchased from Gushen Biotechnology Group Co., Ltd (Shandong, China). Alcalase 2.4 L (196,654 ± 1924 U/mL) was provided by Novozymes Biotechnology Co., Ltd. (Shenyang, China), and SDS-PAGE Gel Kit was purchased from Beijing Solarbio Science & Technology Co., Ltd. (Beijing, China). All other chemicals were of analytical grade and obtained commercially.

### Sample preparation

#### Determination of SPI composition

The composition of the SPI was determined by following the standard methods put forth by the national food safety standards. The moisture content of the SPI was determined by GB-5009.3-2016, protein content by GB-5009.5-2016, fat content by GB-5009.6-2016, and ash content by GB-5009.4-2016.

#### Preparation and limited enzymatic hydrolysis of SPI

The preparation and limited enzymatic hydrolysis of SPI were performed according to the method of Adlernissen (1986), with a slight modification. The SPI solution was prepared by adding distilled water to a final protein concentration of 5% (w/w). After magnetic stirring at room temperature for 1 h, 0.5 mol/L NaOH was added to adjust the solution to pH 8.0. The solution was incubated at 55 °C to start the hydrolysis reaction by adding alkaline protease, and the pH of the solution was maintained constant during hydrolysis by the addition of 0.5 mol/L NaOH using the pH-stat method. The degree of hydrolysis (DH) was calculated by Eq. (1):

1 $${\text{DH}}\left( \% \right) = \frac{{V_{{{\text{NaOH}}}} \times N_{{{\text{NaOH}}}} }}{{\alpha \times M_{p} \times h_{{{\text{tot}}}} }} \times 100\% ,$$

where V_NaOH_ is the amount of alkali consumed (mL), N_NaOH_ is the molarity of alkali (mol/L), *α* is the calibration factor for pH-stat (*α* = 0.4636), *M*_*p*_ is the mass of the SPI (g), and *h*_tot_ is the number of peptide bonds; that is, 7.78 mmol/g protein.

After hydrolysis, the enzyme was inactivated by heating at 90 ℃ for 5 min. After cooling to 30 ℃, the solution was homogenized by Ultra-Turrax T25 (Guangzhou Shenhua Biotechnology Co., Ltd., China) at high speed for 2 min at 13,000 r/min.

#### Drying of soy protein isolate hydrolysate (SPH) solutions

Spray drying of the SPH solution was carried out using the YC-015 bench-top spray dryer (Shanghai Pilotech Instrument & Equipment Co., Ltd., China), where the outlet and inlet temperature were maintained at 70 ℃ and 160 °C, respectively. The rate of the peristaltic pump was maintained at 14 r/min (Joshi et al. 2011).

### Sodium dodecyl sulfate-polyacrylamide gel electrophoresis (SDS-PAGE)

SDS-PAGE was performed using 12% separating gels and 4% stacking gels. A mixture of sample solutions (with an SPI concentration of 10 mg/mL) and loading buffer were heated in boiling water for 5 min, then a volume of 10 µL (μL) was loaded per well and electrophoresis was carried out at 120 V for separation. After electrophoresis, the gels were removed, fixed, and stained with the staining solution (Coomassie brilliant blue 0.125%, ethanol 25%, acetic acid 8%) for the visualization of protein. Finally, destaining was conducted in methanol–water–acetic acid solution (25:67:8, v/v) (Matsumoto et al. 2019).

### Surface hydrophobicity (*H*_0_)

The *H*_0_ of SPH was determined using 1-anilinonaphthalene-8-sulfonic acid (ANS) probes according to a modificatory method by Kato and Nakai (1980). Protein dispersions (1 mg/mL) in 0.01 mol/L phosphate buffer (pH 7.0) were stirred for 1 h at 25 °C and centrifuged at 4000×*g* for 30 min. The protein contents of the supernatant were determined according to Lowry et al. (1951). The supernatant was diluted with the same buffer to keep the protein concentration within the range of 0.005–0.05 mg/mL. Then, 50 μL of ANS (8.0 mmol/L in the same buffer) was added to 4 mL of SPH solution. The fluorescence intensity was measured with a Hitachi FL-2500 fluorescence spectrometer. Excitation and emission wavelengths were set at 365 and 484 nm, respectively, and the excitation and emission slit widths were both 5 nm. The initial slope of the fluorescence intensity vs. protein concentration plot (calculated by linear regression analysis) was used as the index of protein hydrophobicity.

### Fourier transform infrared spectroscopy (FTIR)

According to Zhang et al. (2015a), the Fourier transform infrared spectra of the specimen were evaluated by a Vector 22 Fourier transform infrared spectrometer (Bruker Optics Co., Ltd., Germany). Each spectrum was the cumulative result of 16 scans with a resolution of 4 cm^−1^ from 4000 to 500 cm^−1^ at 25 °C. OMNIC software was used to fit the infrared spectra, and the secondary structure of the specimen was calculated based on its amide I.

### Particle size distribution

The particle size distribution of the SPH sample was determined using a laser particle size distribution instrument (Bettersize Instruments Co., Ltd., China). The SPH sample was dispersed in phosphate buffer to obtain a 1% protein solution. It was then concussion mixing using a laboratory vortex oscillator (Kylin-Bell Lab Instruments Co., Ltd., China). After adding the solution to a colorimetric dish containing double distilled water (DWW), when the refractive index of the solution system reached 34%, the particle size distribution was measured.

### Scanning electron microscopy (SEM)

The surface morphologies of SPH powders were studied by observation under a SU1510 scanning electron microscope (Hitachi, Ltd., Japan) at an accelerating voltage of 5 kV. The samples were sputter-coated with a layer of gold prior to having captured the images.

### Determination of functional properties

#### Analysis of emulsifying properties

Both the emulsifying activity index (EAI) and emulsion stability index (ESI) were determined by the turbidimetric method (Jiang and Zhao 2011). Emulsions were prepared by using 16 mL of SPH solution of 1 mg/mL mixed with 4 mL of refined soybean oil homogenized at 10,000 r/min. The resulting emulsion (50 µL) was immediately pipetted from the bottom of the container into 5 mL of 0.1% (w/v) sodium dodecyl sulfate (SDS) solution. After whirlpool oscillation, absorption values at 0 and 10 min were measured at 500 nm. The EAI and ESI were calculated by Eqs. (2) and (3), respectively:

2 $${\text{EAI}}\;\left( {{\text{m}}^{2} /{\text{g}}} \right) = \frac{{2 \times 2.303 \times A_{0} \times {\text{dilution}}}}{{C \times \left( {1 - \Phi } \right) \times 10^{4} }},$$

3 $${\text{ESI}}\;\left( \% \right) = A_{10} /A_{0} \times 100\% ,$$

where *C* is protein concentration (g/mL) before emulsification; *Ф* is oil volume fraction (v/v) of emulsion (*Ф* = 0.2 here), dilution = 250; and *A*_0_ and *A*_10_ represent the absorbance at time 0 and after 10 min at 500 nm, respectively.

#### Analysis of foaming properties

The foaming properties were evaluated according to the method published by Zhang et al. (2015b), with slight modifications. Protein dispersions (1% w/w, 100 mL) were freshly prepared and homogenized at 10,000 r/min for 2 min. The obtained foam and solution were then jointly transferred to a 250-mL glass cylinder. The volumes of the foam portion were recorded at 0 min to determine foam capacity and at 30 min to determine foam stability. Values for the foaming capacity (FC) and foaming stability (FS) were then calculated by Eqs. (4) and (5), respectively:

4 $${\text{FC}}\;\left( \% \right) = \frac{{V_{0} - V}}{V} \times 100\% ,$$

5 $${\text{FS}}\;\left( \% \right) = \frac{{V_{30} - V}}{{V_{0} - V}} \times 100\% ,$$

where *V* is the volume of protein dispersion before homogenization, and *V*_0_ and *V*_30_ represent the volume of foam portion at 0 and at 30 min, respectively.

### Statistical analysis

Results were presented as the mean ± standard deviation for three replicates. Data analysis was performed using a one-way analysis of variance (ANOVA) with the least significant difference test for determining significance using the standard of *p* < 0.05. Statistical analyses were conducted using Origin 8.0 software. Significance of effects can be found only through statistical analysis methods (Fegade et al. 2013).

## Results and discussion

### Protein composition

The contents of the different components of SPI are shown in Table 1. In all cases, the content of protein exceeded 90% (dry weight) and, correspondingly, all other components obtained values lower than 10%.

**Table 1:** Components of SPI

Components	Content (%)
Water	7.26 ± 0.045
Protein	87.48 ± 0.950
Ash	4.76 ± 0.061
Fat	0.13 ± 0.002

Each value represents the mean ± SD (*n* = 3)

### SDS-PAGE analysis

**Fig. 1:**
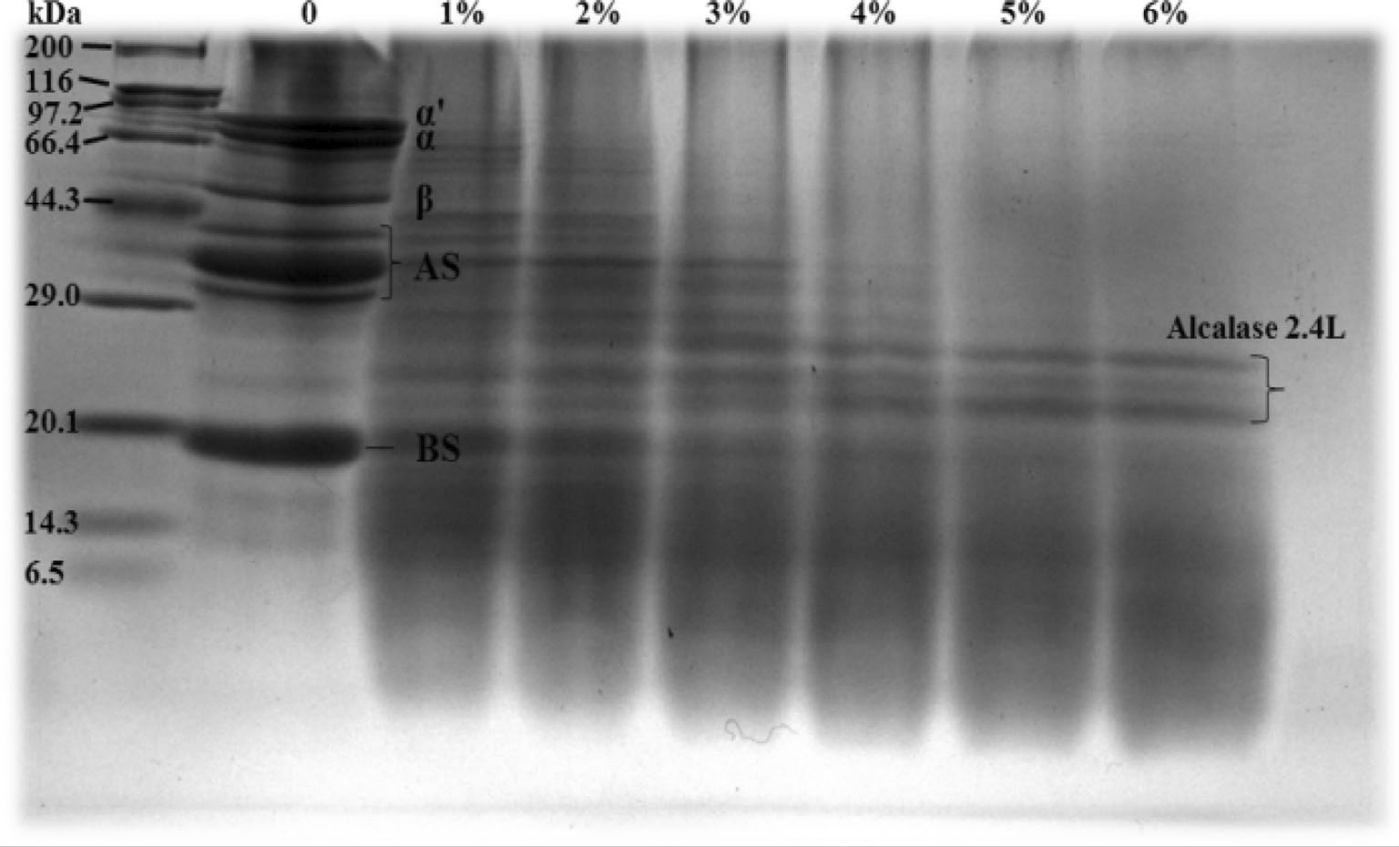
The representative SDS-PAGE patterns of hydrolysates subjected to alcalase at DH of 0–6%, respectively. *BS* basic subunits; *AS* acidic subunits. α, α′ and β are subunits from β-conglycinin

To detect the structural changes of SPH after undergoing the combined treatment of alkaline protease and high-speed shearing homogenization, SDS-PAGE was used to examine the subunit compositions of SPH. Figure 1 shows the protein profile of SPI. At a 0% DH, it contained intact β-conglycinin subunits (*α*, *α*′, and *β*) and glycinin (both the acidic and basic subunits) as measured by SDS-PAGE analysis (Chen et al. 2019). Changes in the subunit ratio of the hydrolyzed protein were expressed, and this was according to the different molecular weights of the protein subunits as well as the migration of the bands generated by the hydrolyzed subunits in the gel electrophoresis diagram. As shown in Fig. 1, the native SPI primarily comprised five subunits, and the content of these five subunits changed significantly following hydrolysis. Compared with unhydrolyzed SPI, subunit structures at each DH were destroyed. Among these, intact subunit structures were significantly reduced at 1% and 2% DH, but the five subunit structures of the 7S globulin and 11S globulin were still clearly visible without their complete degradation. However, when the DH exceeded 3%, the subunit structure of the 7S globulin was almost completely degraded. This indicated the whole degradation of the protein into hydrophilic and amorphous peptides, consistent with previous studies (Chen et al. 2019; Shen et al. 2020). Thus, hydrolysis by alkaline protease with high-speed shearing homogenization significantly degraded the primary structure of SPI, thereby greatly reducing the molecular weight of proteins.

### *H*_0_ analysis

**Fig. 2:**
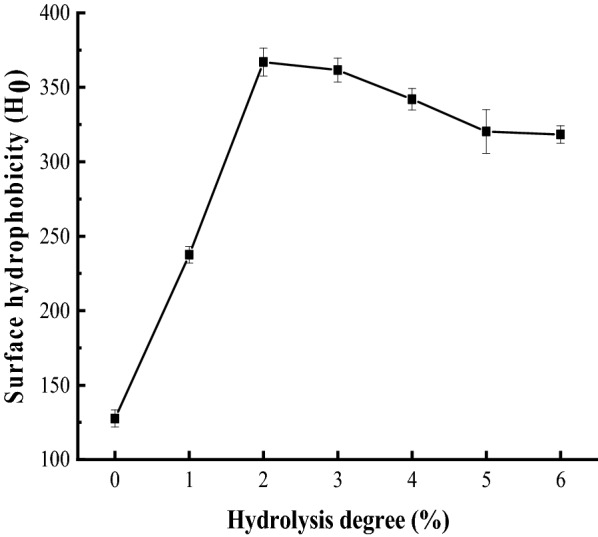
Surface hydrophobicity of SPH at 0–6% DH, respectively

As one of the most important indicators for evaluating changes in protein conformation (Yan et al. 2021), the *H*_0_ of a protein indicates the number of hydrophobic groups present on the surface of the protein molecule. Results depicted in Fig. 2 indicate that a degree of up to 2% hydrolysis caused a significant increase in the *H*_0_, while *H*_0_ began to decline gradually after this threshold. According to Seguracampos et al. (2012), such modest hydrolysis treatment can hydrolyze SPI into small peptide chains, subsequently exposing the hydrophobic groups embedded within the protein molecules, thus significantly increasing hydrophobicity. Thus, characterized by increases in the DH, one possible reason for the increased *H*_0_ following hydrolysis is the exposure of hydrophobic amino acids embedded in the protein. However, higher levels of hydrophobic exposure may also promote protein aggregation, thereby resulting in a decrease in surface *H*_0_ (Ma et al. 2018). Previous studies have reported similar trends (Yuan et al. 2012); limited proteolysis treatment induced the increased release of hydrophobic residues from protein molecules. Homogenization treatments have also been shown to reduce surface *H*_0_ due to the formation of aggregates caused by hydrophobic interactions (Cha et al. 2019).

### Effects of hydrolysis on the secondary structures of SPH

**Fig. 3:**
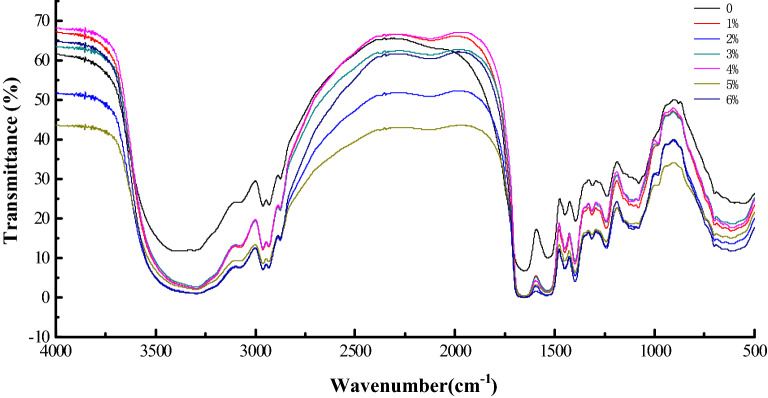
Fourier transform infrared spectroscopy of SPH at 0–6% DH, respectively

The secondary structures of SPH were evaluated by FTIR in the far-infrared region (4000–500 cm^−1^) (Fig. 3) for SPH hydrolyzed by alkaline protease at the DH of 0%, 1%, 2%, 3%, 4%, 5%, and 6% (Table 2). The polypeptide and protein repeat units gave rise to nine characteristic IR absorption bands, namely: amides A, B, and I–VII (Guo et al. 2017). The most sensitive spectral region to the protein secondary structural components is that of amide I, and this was mainly from the C=O stretching vibration of the polypeptide backbone (Akyuz et al. 2018). One of the major factors responsible for the conformational specificity of the amide I band is its sensitivity to hydrogen bonding and the change of secondary structures of proteins mainly depends on hydrogen bonds. Thus, FTIR can sensitively reflect the change in peptide chain structures, as was done with secondary structures in the present study (Hu et al. 2016). As shown in Fig. 3, changes in the FTIR spectra of SPH were observed through characteristic shifts in some band frequencies. Overall, the absorption bands of SPH were markedly different before treatment.

**Table 2:** Secondary structure content of SPH sample

Hydrolysis degree (%)	α-Helix (%)	β-Sheet (%)	β-Turn (%)	Random coil (%)
0	11.83 ± 0.17^a^	32.56 ± 0.55^a^	46.43 ± 0.10^a^	9.21 ± 0.10^a^
1	10.84 ± 0.38^ab^	30.82 ± 0.19^b^	39.36 ± 0.63^b^	12.25 ± 0.27^b^
2	12.58 ± 0.29^ac^	21.40 ± 0.32^c^	36.24 ± 0.16^bc^	21.57 ± 0.85^c^
3	11.94 ± 0.24^a^	21.68 ± 0.44^c^	41.43 ± 0.38^bd^	18.05 ± 0.55^d^
4	11.95 ± 0.08^a^	21.45 ± 0.23^c^	39.35 ± 0.97^bd^	19.26 ± 0.38^d^
5	12.82 ± 0.20^ac^	20.74 ± 0.45^c^	41.47 ± 0.60^bd^	18.31 ± 0.23^d^
6	25.83 ± 0.93^d^	23.78 ± 0.28^d^	17.96 ± 0.28^e^	12.70 ± 0.21^b^

The results were expressed as the mean ± SD (*n* = 3). Values with different superscript letter are significantly different at *p* < 0.05

The secondary derivative spectra of SPH were fitted by the Gaussian peak to show characteristic peaks including those of the α-helix (1650–1658 cm^−1^), β-sheet (1610–1640 cm^−1^), β-turn (1660–1670 cm^−1^), and random coil (1640–1650 cm^−1^) (Achouri et al. 2012). The areas of all bands were then assigned to a given secondary structure, summed up, and divided by the total area to obtain the contribution of each element. As shown in Table 2, compared with the native SPI, the content of the β-sheet and β-turn structures dramatically decreased (*p* < 0.05), while that of the random coil significantly increased (*p* < 0.05). With only 6% hydrolysis, the content of α-helix structures increased significantly (*p* < 0.05). Moreover, initial hydrolysis-assisted homogenization had a significant effect on secondary structures such as the β-sheet, β-turn, and random coil. Conversely, under the condition of 3–5% hydrolysis, there were no significant changes in the secondary structures. When the degree of hydrolysis reached 6%, compared to the 5% hydrolysis, contents of the α-helix structure increased by 13.01%, that of the β-sheet increased by 3.04%, that of the β-turn decreased by 23.51% and that of random coil decreased by 5.61% (Fig. 3). This observation indicates that the random coil and β-turn structures were transformed into α-helix and β-sheet structures.

The results showed that different degrees of hydrolysis by alkaline protease and high-speed shear homogenization treatment significantly influenced the secondary structures of SPH and that this was strongly associated with subunit composition. The α-helix is believed to be maintained by intramolecular hydrogen bonds while the β-sheet is supported by hydrogen bonds between peptide chains (Tang and Ma 2009). The changes in secondary structures suggested that hydrolysis with high-speed shear homogenization increased random coil structures and decreased β-turn structures, with almost all of the changes occurring on the surface of protein molecules (Table 2). It was found that the limited enzymatic hydrolysis of less than 6% combined with high-speed shear homogenization destroyed the hydrogen bonds between the peptide chains while having little effect on intramolecular hydrogen bonds. At 6% hydrolysis, a stronger aggregation force formed more stable structures while decreasing both random coil and β-turn structures. However, a previous study also reported a significant decrease in α-helix, β-sheet, and random coil structures, as well as an increase in β-turn structures with the pretreatment of high-pressure homogenization (HPH) before the hydrolysis of SPI (Zhao et al. 2018). The most probable reason for the diversity in these results may be that the high-pressure homogenization pretreatment caused the initial decomposition of protein molecules, thereby resulting in more β-turn structures.

### Particle size distribution

**Fig. 4:**
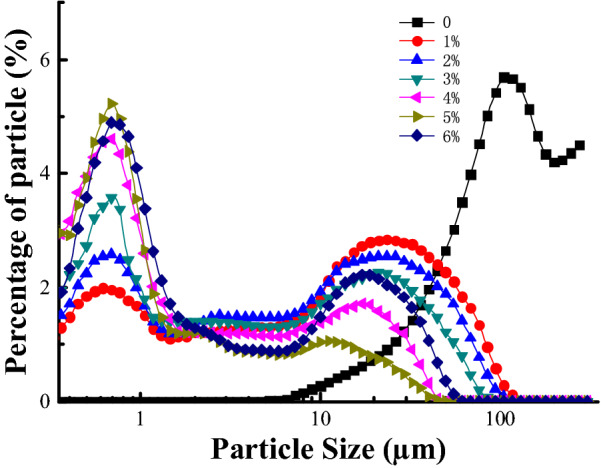
Particle size distribution of SPH at 0–6% DH, respectively

As shown in Fig. 4, the natural SPI particle size distribution was bimodal, and after limited enzymatic hydrolysis with high-speed shear homogenization, it still showed this bimodal morphology, with the main two peaks residing near 0.7 and 20 μm, respectively. With the increase in the DH, the morphology of these double peaks for SPH particle size gradually merged into a single peak. Meanwhile, the peak position for large particle size gradually shifted to that of small particle size. This showed that the proteins were more evenly dispersed in the solution system and that the protein solution became increasingly stable. When the degree of hydrolysis reached 6%, the content of SPH containing large particle sizes increased. This result might be attributed to the increased formation of stable aggregates with smaller particle sizes (Huang et al. 2019). The median diameter (D50) of the SPH sample at 0–6% DH is shown in Table 3.

**Table 3:** Median diameter (D50) of SPH sample

DH (%)	Particle size of SPH sample (μm)
1	10.750 ± 0.345^a^
2	5.623 ± 0.140^b^
3	4.008 ± 0.104^c^
4	2.158 ± 0.110^d^
5	1.532 ± 0.199^d^
6	8.153 ± 0.333^e^

The results were expressed as the mean ± SD (*n* = 3). Values with different superscript letter are significantly different at *p* < 0.05

### SEM observation

**Fig. 5:**
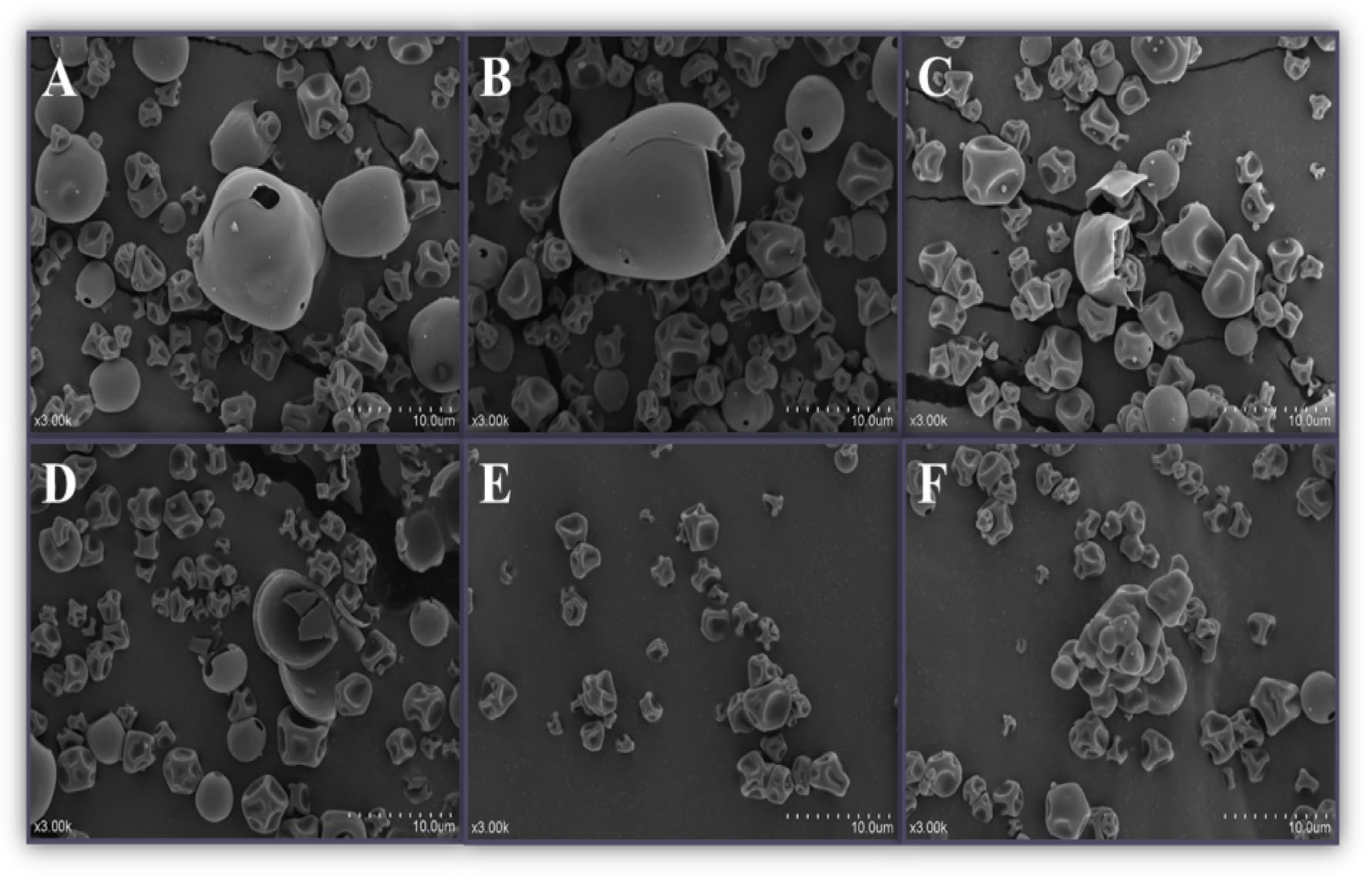
SEM micrographs of SPH. The letters **A**–**F** represent 1–6% DH, respectively

To further investigate the effects of limited enzymatic hydrolysis combined with high shear homogenization on the aggregation state of soybean protein isolate molecules, the macrostructure of SPH was observed by scanning electron microscopy (SEM). As shown in Fig. 5, varying degrees of hydrolysis caused varying degrees of damage to SPI, and the surface of spray-dried sample particles was mostly concave. Some of the visible SPI molecules inside were hollow structures. According to Buma and Henstra (1971), the formation of this kind of structure is likely to be caused by the uneven shrinkage of particles formed by protein and water during spraying, thus leading to the appearance of hollow structures in some SPH that have not yet contracted after water loss. Furthermore, with increases in the DH, the finer structures became observable, and when the degree of hydrolysis reached 6%, some stable aggregates of SPH were also observed from the images. This indicated that hydrolysis with homogenization treatment could destroy the globular structure of proteins and that the smaller particles would lead to further aggregation (Huang et al. 2019). Interestingly, this trend is in agreement with the results of the previous measurement of the particle size distribution (Fig. 4). It also indicated that smaller protein particles were more likely to promote aggregation.

### Functional properties of SPH

#### Emulsifying properties of SPH

**Fig. 6:**
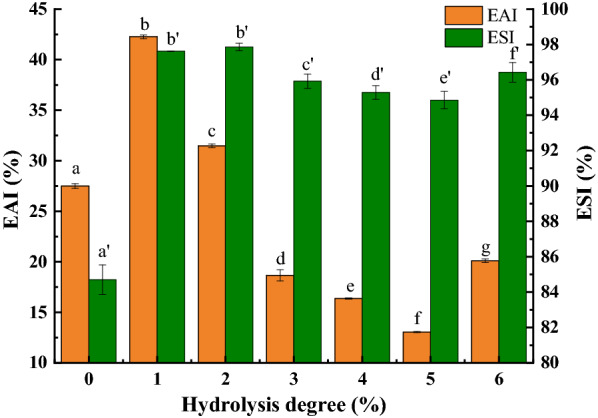
Emulsification properties of SPH at 0–6% DH, respectively. *EAI* emulsifying activity index; *ESI* emulsion stability index. Values with different superscript letter are significantly different *p* < 0.05. a–g is used for EAI and a′–f′ is used for ESI

Emulsifying properties indicate the ability of a protein to absorb to oil–water interfaces and can be evaluated via the protein’s emulsifying activity index (EAI) and emulsion stability index (ESI). The effect of limited enzymatic hydrolysis with high-speed shear homogenization on the emulsifying properties of SPI is shown in Fig. 6. Compared with SPI without hydrolysis treatment, a notable increase in the EAI of SPH was observed. When the degree of hydrolysis reached 1%, the highest value was obtained for the EAI. With the increase in the DH, the emulsifying activity decreased, but there was a rebound until 6% hydrolysis. This trend, whereby the emulsifying activity of SPH first increased and then decreased, might be attributed to changes within the protein surface structure (Jiang et al. 2009). Specifically, hydrolysis may have destroyed the spherical structure of the protein, exposing more hydrophobic residues (Fig. 5), which then improved the emulsifying ability of SPH, while higher DH resulted in a reduction of the flexibility of SPH, which in turn reduced its emulsifying ability. If the interactions between protein molecules were too strong, this would be detrimental to the emulsification ability of protein (Ma et al. 2020b). With increasing DH, the molecules were more refined, and this enhanced intermolecular interactions. The hydrogen bonds that maintained the internal structure of SPH were gradually destroyed, which also made it difficult to form a stable protective layer on the surface of oil droplets.

As shown in Fig. 6, the ESI of the dispersion prepared using SPH samples was significantly enhanced. Generally, the ESI of protein emulsions are related to their particle size (Yang et al. 2018), and the particle size distribution of SPH in our study has confirmed this (Fig. 4). Additionally, our results show that the ESI of SPH emulsions decreased when the DH exceeded 2% and that when the DH reached 6%, there was a slight rebound effect. The decrease of the ESI in the SPH emulsion might be due to a decrease in the molecular flexibility of the proteins (Molina et al. 2001).

Thus, the functional properties of proteins were essentially determined by their conformations and surface properties. Based on the above results, hydrolysis and protein aggregation both had a complex impact on the flexibility, hydrophilicity, and hydrophobicity of SPH molecules during treatment involving hydrolysis and homogenization, which consequently impacted the EAI and ESI.

#### Foaming properties of SPH

**Fig. 7:**
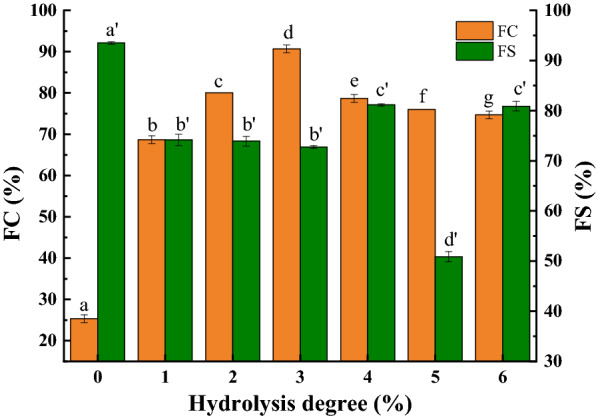
Foaming properties of SPH at 0–6%DH, respectively. *FC* foaming capacity; *FS* foaming stability; values with different superscript letter are significantly different *p* < 0.05. a–g is used for FC and a′–d′ is used for FS

Foaming properties are another important indicator of the quality of protein as a surfactant. Stable foaming is required so that the protein possesses moderate mechanical strength and viscosity characteristics in order to maintain the formed network structure. So, the flexibility of protein molecules and the strength of the connection between peptides have become key factors in determining foaming stability. As shown in Fig. 7, compared with native SPI, the foaming capacity (FC) of SPH by limited enzymatic hydrolysis combined with high shear homogenization was significantly improved. In addition, as the DH increased, the FC of the SPH emulsion increased (at 1–3% DH) and then decreased (at 4–6% DH). However, compared with the SPI sample, as the DH increased, SPH exposed more hydrophobic groups and had a looser spherical conformation (Table 2) as well as a higher proportion of small molecule peptides (Fig. 1), which is beneficial to the formation of a liquid film at the interface between air and water. Therefore, the reduced molecular weight would be assumed to make them more flexible. Moreover, the speed of SPH molecular transferring to the air–water interface was faster and higher in efficiency in reducing surface tension (Mao and Hua 2012). Thus, hydrolysis with high-speed shear homogenization treatment could significantly enhance the foaming capacity. Based on the results of the particle size distribution, with the increase in the DH, shorter peptide chains were formed, hence resulting in the weakening of the liquid membranes which were formed on the surface of the liquid by unfolding peptide chains (Fig. 4). The weakening of these liquid membranes might have had an inhibitory effect on the foaming properties of the SPH solution.

The results of foaming stability (FS) showed that the FS of SPH was reduced compared with that of the native SPI. Specifically, the FS was the lowest for SPH with 5% DH, which might have been due to the smaller peptide segments in the hydrolysate causing the liquid membranes to be fragile, which weakened the capability of encapsulating the foam within liquid membranes. Compared with the SPI samples, the formation of more polypeptides in the SPH samples increased the charges of protein molecules, which impeded the adsorption of protein molecules onto the surface of foam, thereby resulting in the decrease in foam stability (Matsumiya and Murray 2016).

## Conclusion

In our study, treatment with hydrolysis with high-speed shear homogenization exhibited a significant influence on the structural and functional properties of SPI. The results showed that hydrolysis had a certain destructive effect on the primary structure of SPI, and the degree of destruction of 7S and 11S globulins gradually increased with increasing DH. The results of FTIR also showed that limited enzymatic hydrolysis combined with high shear homogenization had a marked effect on the hydrogen bonding present among peptide chains, thereby resulting in a reduction of the contents of β-sheet and β-turn structures. Restriction hydrolysis significantly reduced the protein particle size in the protein solution system, especially when the DH reached 5%. Furthermore, results for hydrolyzed protein processing characteristics showed that mild hydrolysis could significantly reduce the particle size of protein particles to improve their emulsification properties. However, with the increase in the DH, the flexibility of protein molecules decreased, the interaction between molecules strengthens and the emulsification significantly decreased. Compared with SPI, SPH displayed significantly improved foaming properties due to enhanced hydrophobicity and the destruction of protein molecular structures, and we obtained the key result of the worst foaming stability when the degree of hydrolysis was 5%. Therefore, the results of the present work provide a theoretical basis for the further study of micronized proteins, and can also be used in other food processing areas, such as in the research and development of fat replacements and soy peptides. They also comprise an effective means to modify the functionality of globular proteins. Therefore, the combination of alkaline protease hydrolysis and high-speed shearing homogenization represents an effective method by which to improve the functional properties of SPI.

## Data Availability

All data generated or analyzed during this study are included in this published article.
